# Predicting protein model correctness in *Coot* using machine learning

**DOI:** 10.1107/S2059798320009080

**Published:** 2020-07-27

**Authors:** Paul S. Bond, Keith S. Wilson, Kevin D. Cowtan

**Affiliations:** a Department of Chemistry, University of York, York YO10 5DD, United Kingdom

**Keywords:** structure solution, software, model building, validation, machine learning, *Coot*

## Abstract

Manually identifying and correcting errors in protein models can be a slow process, but improvements in validation tools and automated model-building software can contribute to reducing this burden. This article presents a new correctness score that is produced by combining multiple sources of information using a neural network. The residues in 639 automatically built models were marked as correct or incorrect by comparing them with the coordinates deposited in the PDB. A number of features were also calculated for each residue using *Coot*, including map-to-model correlation, density values, *B* factors, clashes, Ramachandran scores, rotamer scores and resolution. Two neural networks were created using these features as inputs: one to predict the correctness of main-chain atoms and the other for side chains. The 639 structures were split into 511 that were used to train the neural networks and 128 that were used to test performance. The predicted correctness scores could correctly categorize 92.3% of the main-chain atoms and 87.6% of the side chains. A *Coot* ML Correctness script was written to display the scores in a graphical user interface as well as for the automatic pruning of chains, residues and side chains with low scores. The automatic pruning function was added to the *CCP*4*i*2 *Buccaneer* automated model-building pipeline, leading to significant improvements, especially for high-resolution structures.

## Abstract 2

Two neural networks were trained to predict the correctness of protein residues by combining multiple validation metrics in *Coot*. Using the predicted correctness to automatically prune models led to significant improvements in the *Buccaneer* pipeline.

## 1. Introduction   

Manual completion of a model is a very time-consuming step in macromolecular structure solution. Initial models from homologues or from automated model-building programs will contain errors that must be identified and corrected. The primary method for identifying errors is visual examination of the model, the 2*mF*_o_ − *DF*_c_ map and the *mF*_o_ − *DF*_c_ map by the crystallographer, using a model-building program such as *Coot* (Emsley & Cowtan, 2004 ▸; Emsley *et al.*, 2010 ▸). Errors can often be identified by visual examination alone. However, other validation metrics become more important in guiding decisions when the density is less obvious, for example in less ordered regions or lower resolution structures. *Coot* provides validation tools to identify Ramachandran outliers, unusual rotamers and other potential errors, as well as an interface to some tools from *MolProbity* (Williams *et al.*, 2018 ▸). The job of the crystallographer is to combine all of these sources of information and decide whether the model is acceptable or whether it needs to be changed. The work presented here aims to emulate this decision-making process by using machine learning to predict the correctness of protein residues. Machine learning is well suited for this problem as expected patterns in the data are not written into the model in advance but can be found through analysis of the training data. A recent example from the field of crystallography is the use of initial data-processing statistics to predict whether the data are suitable for successful structure determination through SAD/MAD phasing (Vollmar *et al.*, 2020 ▸).

The correctness of a model is not something that is easy to define. If the coordinates of an atom are altered gradually, there is no definitive point at which the position becomes correct. The model needs to fit both the experimental data and previously acquired knowledge of atomic structures, especially at lower resolution when it is not possible to distinguish individual atomic peaks. In this space it is likely there are multiple local minima, the positions of which will vary depending on the refinement procedure. However, alternate conformations aside, usually only one minimum is considered to be correct within an individual refinement procedure.

Predicting the correctness of residues can be formulated as a supervised machine-learning problem, where each data point has several feature attributes that are used to predict another target attribute. In this application, a data point is a residue, the features are pieces of information about the residue, for example the Ramachandran score and a score of the fit to density, and the target is correctness. The prediction could be performed using either classification, where each residue is labelled as correct or incorrect, or regression, where a numerical correctness score is assigned. It was decided to use regression as the score would be useful for graphical validation tools and for automated procedures to select badly scoring residues at various thresholds.

The amount of manual model-building work that needs to be performed can be drastically reduced by having better automated model-building programs that lead to models with fewer errors. *Buccaneer* (Cowtan, 2006 ▸, 2008 ▸) is a fast model-building program that works well at a range of resolutions and is distributed with the *CCP*4 software suite (Winn *et al.*, 2011 ▸). It does not perform any global refinement of coordinates or *B* factors, so it is most effective when combined with a refinement program such as *REFMAC* (Murshudov *et al.*, 2011 ▸; Kovalevskiy *et al.*, 2018 ▸) in an iterative pipeline. The refinement program improves the model geometry and fit to density and produces an updated map that can be passed to the next building cycle. There are *Buccaneer* pipelines available in *CCP*4*i* (Potterton *et al.*, 2003 ▸) and *CCP*4*i*2 (Potterton *et al.*, 2018 ▸). *Buccaneer* is also used in other pipelines such as *CRANK* (Ness *et al.*, 2004 ▸; Pannu *et al.*, 2011 ▸), *CAB* (Burla *et al.*, 2018 ▸) and *CCP*4*Build*, which is a new model-building pipeline available in *CCP*4*Cloud* (Krissinel *et al.*, 2018 ▸).

It has been observed that although *Buccaneer* is good at building complete structures at low resolution, it can build more incorrect residues than other programs (van den Bedem *et al.*, 2011 ▸; Alharbi *et al.*, 2019 ▸). The incorrect residues are mostly small unsequenced chains built into the solvent that need to be removed by the user at the end of the pipeline. There are already some existing steps within *Buccaneer* for removing chains: the filter step removes chains shorter than six residues and the pruning step solves clashes between chains by truncating the chain with the most unsequenced residues or the shorter chain. However, if the chain contains at least six residues and does not overlap with another chain, then it will be kept. It would also be useful to have a method for deleting individual residues and side chains identified as incorrect. Errors such as peptide bonds that need flipping and side chains built with the wrong rotamer are not uncommon. If pruning these errors is followed by refinement, then the resulting likelihood-weighted maps will be less biased towards the error and future automated building cycles are more likely to correct the issue. A pruning step has already been implemented in *CCP*4*Build* that uses real-space difference density *Z*-scores (RSZDs) from *EDSTATS* to identify residues and side chains to delete. The RSZD metric is calculated separately for main-chain atoms and side chains and is useful for determining how accurately parts of a structure fit the electron density, but the calculation can be slow for high-resolution structures. A new pruning step is presented here that uses the machine-learned correctness scores to delete whole chains, individual residues and side chains. We show that this pruning step enhances the ability of the *Buccaneer* pipeline to self-correct mistakes and produce better models that need less manual correction.

## 2. Methods   

Calculations were performed on a Scientific Linux 7.7 server with two AMD EPYC 7451 CPUs and 256 GB RAM. Programs were sourced from *CCP*4 7.0.076 (Winn *et al.*, 2011 ▸).

### 2.1. Structure-set curation   

A program was written for choosing sets of target structures and creating molecular-replacement models using existing structures in the PDB (Berman *et al.*, 2000 ▸). The goal was to choose diverse, good-quality target structures that cover a range of resolutions and to produce a range of molecular-replacement models, some leading to good-quality phases and some leading to poor-quality phases. Using this program, 1800 target structures at 1–3.5 Å resolution were chosen with 11 183 molecular-replacement models between them. This set was reduced by choosing a subset of the target structures with only one molecular-replacement model per structure. Two reduced sets were created: a full reduced set with 1351 structures at 1–3.5 Å resolution with a wide range of initial phase qualities and an easy reduced set with 639 structures at 1–2.5 Å resolution with only good-quality phases. The program and structure sets are documented in detail in the supporting information and are available to other developers.

### 2.2. Neural network target   

For the 639 structures in the easy reduced set, models automatically built with the *CCP*4*i* *Buccaneer* pipeline were used to provide examples of both correct and incorrect residues. Refined versions of the models deposited in the PDB were used as references that are assumed to be wholly correct. As detailed in the supporting information, target structures were only chosen if they had good overall quality indicators, *i.e.* *R*_free_, clashscore (Williams *et al.*, 2018 ▸) and percentage outliers, so only a small minority of residues should have errors. The target correctness values of residues were assigned by comparing them with the reference structure. An alternative would be to label residues manually, which could be more accurate but would be very time-consuming and many samples are needed for higher coverage of the feature space. The *Buccaneer* models were first moved onto the reference using *CSYMMATCH*, which searches for the best fit using symmetry operations and allowed origin shifts, and refined again using *REFMAC*. For an individual residue, if all of the main-chain atoms, including the C^β^ atom, are within 1 Å of an equivalent atom in the reference, then the main chain of that residue is given a correctness score of 1. However, if one of the atoms is more than 1 Å away from the reference then the main chain of the residue is given a correctness score of 0. The same calculation is performed for the side-chain atoms from the γ position onwards. Asparagine, glutamine and histidine have side chains that still fit the density well if the terminal χ angle is rotated by 180°, so these are classed as correct if built either way round.

### 2.3. Neural network features   

The features used are summarized in Table 1 ▸. There are 12 features for predicting main-chain correctness and nine features for predicting side-chain correctness. Eight features are used for both but, other than resolution, they are calculated separately for the main-chain atoms (N, C^α^, C^β^, O and C) and side-chain atoms from the γ position onwards. *Coot* 0.8.9.2 was used to calculate all features using functions described in the *Coot* user manual (Emsley, 2020 ▸). Explanations of individual features can be found in Sections 2.3.1–2.3.9.

#### 2.3.1. Map-to-model correlation   

Correlation coefficients were calculated using the *map-to-model-correlation* function. Two different masks were used to calculate this separately for the main-chain atoms (atom mask mode 1) and the side-chain atoms excluding the C^β^ atom (atom mask mode 3).

#### 2.3.2. Density *Z*-scores   

Values of the 2*mF*_o_ − *DF*_c_ (best) density map and the *mF*_o_ − *DF*_c_ (difference) density map were measured at the atomic positions of each atom. The raw map values were normalized by dividing them by the atomic number of the atom, and they were then converted to modified *Z*-scores using (1) (Iglewicz & Hoaglin, 1993 ▸), where 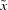 is the sample median:
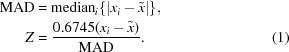
This uses the median of absolute deviations from the median (MAD) as a replacement for standard deviation as it should be more robust in skewed distributions. *Z*-scores were calculated separately for main-chain and side-chain atoms over the whole structure. Three features were used to predict both main-chain and side-chain correctness: the mean best density *Z*-score, the minimum best density *Z*-score and the minimum difference density *Z*-score. In addition, the difference density *Z*-score at the C^α^ position of the next residue was used as a feature to predict main-chain correctness.

#### 2.3.3. *B*-factor *Z*-scores   

Isotropic *B* factors were recorded for each atom, as well as the maximum percentage increase from the *B* factors of bonded atoms. *B* factors and the maximum change in *B* factors were converted to modified *Z*-scores for main-chain and side-chain atoms as described in Section 2.3.2. The maximum *B*-factor *Z*-score and maximum *B*-factor change *Z*-score were used to predict both main-chain and side-chain correctness.

#### 2.3.4. Atom overlap   

To measure the extent to which a residue clashes with its neighbours, a list of atom-overlap volumes was obtained using the *molecule-atom-overlaps* function. This was used to calculate the maximum overlap for the main-chain atoms and side-chain atoms of each residue.

#### 2.3.5. Resolution   

The high-resolution limit of the data does not vary per residue, but it was included as a feature as it should be useful for adjusting the weights of other features.

#### 2.3.6. Ramachandran score   

The main-chain conformation of each residue is assigned a probability based on how often the combination of its φ and ψ angles are observed in high-quality protein structures. This information was obtained using the *all-molecule-ramachandran-score* function, which uses three probability distributions derived from the Top500 database (Lovell *et al.*, 2003 ▸): one for glycine, one for proline and one for other residue types.

#### 2.3.7. Peptide twist   

The twist of a peptide bond was measured as the minimum deviation of the ω angle from either 0° or 180°. For residues connected by two peptide bonds, the largest twist is used.

#### 2.3.8. Pepflip peak   

This is a binary feature that indicates whether there is a positive peak in the difference map at a position that the N or O atoms of a residue could move to if the peptide bond was rotated. A list of positive difference-map peaks was generated using the *map-peaks-around-molecule* function. Each main-chain N and O atom was then examined to see if it could be rotated to any of these peaks by checking the distances and angles between the peak and the main-chain atoms. Initial estimates were made for the r.m.s.d. threshold used for peak picking and acceptable ranges for distances and angles. The estimates were then refined using Nelder–Mead minimization (Nelder & Mead, 1965 ▸) on the full set of 639 structures. The function being minimized was −TP+5FP, where TP is the number of true positives, *i.e.* residues that have a pepflip peak and a main-chain target correctness of 0, and FP is the number of false positives, *i.e.* residues that have a pepflip peak and a main-chain target correctness of 1. With the minimized parameters, only positive difference-map peaks above 4.45 r.m.s.d. were considered. For the peak to be attributed to the O atom, the distance between the peak and the C atom had to be 0.89–2.75 Å, the distance between the peak and the C^α^ atom had to be 1.01–3.71 Å, the distance between the peak and the C^α^ atom of the next residue had to be 1.84–3.87 Å and the angle between the peak, the C atom and the O atom had to be greater than 60.9°. For the peak to be attributed to the N atom the distance between the peak and the C^α^ atom had to be less than 2.09 Å and the distance between the peak and the O atom of the previous residue had to be less than 1.46 Å.

#### 2.3.9. Rotamer score   

These were obtained using the *rotamer-score* function, which uses data from the *MolProbity* Top 500 database (Lovell *et al.*, 2003 ▸). The most commonly observed rotamer is assigned a score of 100. Other conformations are scored relative to this based on their observed frequencies within the database.

### 2.4. Neural network training   

The 639 structures were randomly split, using a 4:1 ratio, into a training set of 511 structures with 305 594 residues and a test set of 128 structures with 76 891 residues. Only residues with side chains longer than C^β^ were used in the side-chain neural network, of which there are 229 967 residues (75.3%) in the training set and 57 522 (74.8%) in the test set. This excludes glycines and alanines, as well as unknown residues that are built as alanine by *Buccaneer*.

The preprocessing and training procedure was the same for both main-chain and side-chain correctness. If a residue had a missing feature, because it depends on neighbouring residues that may not be present, it was assigned the median value of that feature in the training set. The features in the training set were then transformed to have a mean of 0 and a unit variance. The same transform was applied to the features in the test set using the means and standard deviations from the training set.

**Figure 1:**
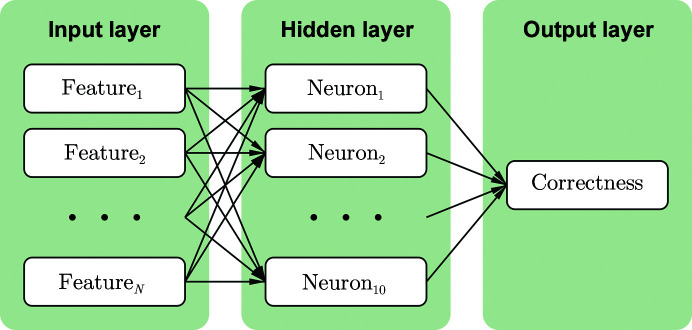
Diagram of the neural network. The input layer contains *N* scaled features (12 for the main-chain network and nine for the side-chain network), the hidden layer contains ten neurons and the output layer contains only one output with the correctness value. Each arrow has an associated coefficient and intercept that are modified during training.

Regression was carried out using a multi-layer perceptron (MLP) neural network from *scikit-learn* version 0.21.2 (Pedregosa *et al.*, 2011 ▸), which trains using back-propagation with the square error as a loss function. Both networks had one hidden layer with ten neurons using the hyperbolic tan function as an activation function, and a single output giving the correctness value without an activation function. Default values were kept for all other parameters, for example the α regularization term was 0.0001 and optimization was carried out using *Adam* (Kingma & Ba, 2014 ▸) for a maximum of 200 iterations. A diagram of the neural network is shown in Fig. 1 ▸ and an equation for calculating Correctness from the input features is shown in (2), where *w_nk_* and *c_nk_* are the coefficient and intercept between Feature_*n*_ and Neuron_*k*_, and *w*_*k*o_ and *c*_*k*o_ are the coefficient and intercept between Neuron_*k*_ and the output node:
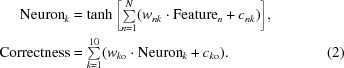



The trained neural networks were scored on both the training and test sets using the coefficient of determination (COD), which assesses the fit between the predicted and target correctness values. The coefficient of determination is usually referred to as *R*2, but this was avoided owing to confusion with the crystallographic *R* factor. It varies between 0, where the model is no better than the mean of the target values, and 1, where the model perfectly predicts all target values. Training was repeated 100 times with different random-number seeds and performance was assessed using the mean and standard error in the COD over the test set. The first trained network, with a random seed of 0, was used as the final predictor. To test whether all of the features should be included in the network, features were removed one at a time and the training repeated, again using 100 different seeds, to establish the change in the COD.

The final predictor was also assessed on its ability to classify residues in the test set by converting the correctness score to a binary class, where a score of ≥0.5 is predicted to be correct. The residues in the test set were then split into true positives (TP) that are actually correct and predicted to be correct, true negatives (TN) that are actually incorrect and predicted to be incorrect, false positives (FP) that are actually incorrect but predicted to be correct, and false negatives (FN) that are actually correct but predicted to be incorrect. Equations (3) to (10) show a number of quality metrics that were derived from these counts.
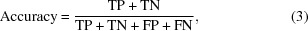


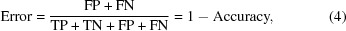


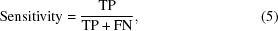


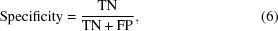


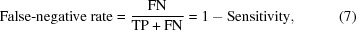


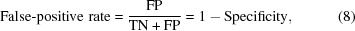


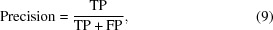


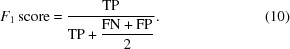



### 2.5. *Coot* ML Correctness script   

A *Coot* ML Correctness script was created that calculates the features and uses the trained neural networks to obtain the main-chain and side-chain correctness scores for each residue. Machine-learning data were incorporated into the script through an object containing the medians for each feature, the means and variances used for scaling features and the coefficients and intercepts used by the neural networks. Running this script creates two new menu items in the *Coot* user interface under the heading ‘ML Correctness’. The first is a graphical user interface (GUI) that has lists of all the residues along with their correctness scores. Clicking on a residue will move the view in the main window to that location. Owing to the time that it takes to calculate some of the features, the GUI does not update as the model changes, but check boxes are provided so the user can keep track of which issues have been addressed.

The second menu item is an automatic pruning function that deletes whole chains, whole residues and side chains with low correctness scores. Whole chains of up to 20 residues in length are deleted if the mean main-chain correctness for that chain is less than 0.2 times the median main-chain correctness in the full structure. Individual residues and side chains are deleted if the main-chain and side-chain correctness scores, respectively, are less than half of the median for the full structure. After the low-scoring residues have been deleted, isolated residues are also removed. A maximum of 20% of the residues or side chains are deleted at each stage. The pruning function is also available via a scripting interface, where it can be called with custom parameters.

### 2.6. *Buccaneer* pipeline   

As described in Section 2.5, the *Coot* ML Correctness script contains an automatic pruning function that deletes chains, individual residues and side chains with low completeness scores. This function was incorporated into two new versions of the *CCP*4*i*2 *Buccaneer* pipeline that are summarized in Table 2 ▸. The chain-pruning pipeline has an additional step that prunes whole chains at the end of each iteration, followed by a further five cycles of refinement using *REFMAC*. The full pruning pipeline also starts each iteration, other than the first, by deleting chains, residues and side chains in the model from the previous cycle, running five cycles of *REFMAC* and passing the updated model and map to *Buccaneer*.

All three pipelines were tested on 867 structures between 1 and 3.5 Å resolution from the full reduced set. The full reduced set contains 1351 cases, but 483 were excluded because the target structures were part of the neural network training set. Another structure, PDB entry 5da8, was excluded because the noncrystallographic symmetry in this case leads to very long run times using the version of *Buccaneer* in *CCP*4 7.0.076; this issue has been addressed in *CCP*4 7.1. The pipelines were run using default parameters starting from the molecular-replacement model.

## 3. Results and discussion   

### 3.1. Neural network training   

The COD for the trained neural network models is shown in Table 3 ▸ for both the training set and the test set. Values are given as the mean with an uncertainty of one standard error after repeating the training 100 times with different random-number seeds. If the COD was much higher for the training set than the test set this could indicate overfitting, but in this case the values for the test set are higher. Overfitting is unlikely owing to the large number of residues and the small size of the neural network, but there could be some differences between the training and test sets depending on the random split of the 639 structures. The COD is lower for the side-chain network, but this is heavily dependent on the proportion of correct residues. The main-chain sets contain a higher proportion of correct examples so a higher COD is expected.

**Figure 2:**
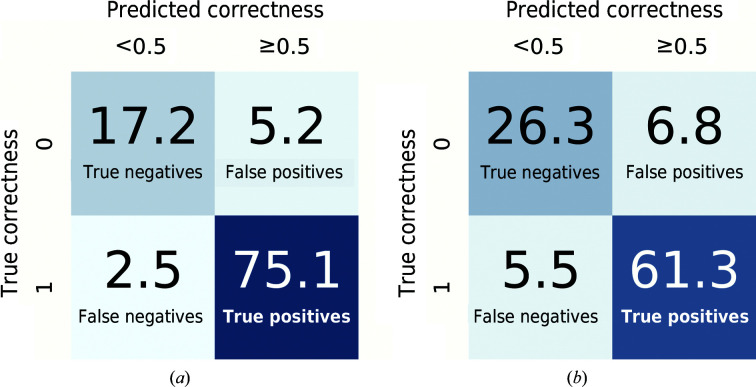
Confusion matrices for (*a*) the main-chain and (*b*) the side-chain network. Values shown are percentages of residues in the test set.

Although regression was used instead of classification, two classes were obtained using a threshold correctness of 0.5. Confusion matrices, which show the relationship between target (true) correctness and predicted correctness, are presented in Fig. 2 ▸. Table 4 ▸ shows various quality metrics derived from the number of true positives, true negatives, false positives and false negatives in the test set. Both networks do a good job at identifying correct residues but are less good at identifying incorrect residues, as shown by the difference in the sensitivity (true-positive rate) and specificity (true-negative rate) or, equivalently, by the false-positive rate being much higher than the false-negative rate. This is a symptom of the training data, especially the main-chain data, containing mostly correct residues, so the networks are more likely to assume that a residue is correct. The correctness threshold of 0.5 could be increased for a higher specificity at the cost of lower sensitivity.

The simplistic method of determining the target correctness needs to be taken into account when comparing the true and predicted correctness values. This was performed by comparing each residue with the deposited structure. If any atom was more than 1 Å away it was marked as incorrect. Firstly, the cutoff was not chosen based on any analysis of existing data. It was just assumed that at both high and low resolution the same conformation is usually closer than 1 Å and different conformations are usually further apart than 1 Å after refinement. Another issue is that not all acceptable conformations will be modelled in the deposited structure, especially for flexible side chains at low resolution, when it is hard to distinguish multiple conformations. In addition, the deposited model may also contain errors. Structures were filtered based on overall quality indicators from the wwPDB validation report, but local problems may still exist. However, the training and test sets are still useful for machine learning, and a larger, noisy data set can even produce a better predictive model than a smaller, less noisy one.

For both neural networks, the input features were removed one at a time and the training was repeated to establish the magnitude and significance of the change in the COD. Table 5 ▸ shows the results for the main-chain features and Table 6 ▸ shows the results for the side-chain features. However, the change in the COD depends both on how much useful information a feature has and how well it correlates with other features. If removing a feature leads to no decrease in the COD then it either does not provide information that is useful for identifying incorrect residues or the information is duplicated in another feature. In either case the feature can be removed. If removing a feature causes a large reduction in the COD then it is both useful and independent. All of the features give a significant reduction in the COD when removed, so they are all providing some useful information.

**Figure 3:**
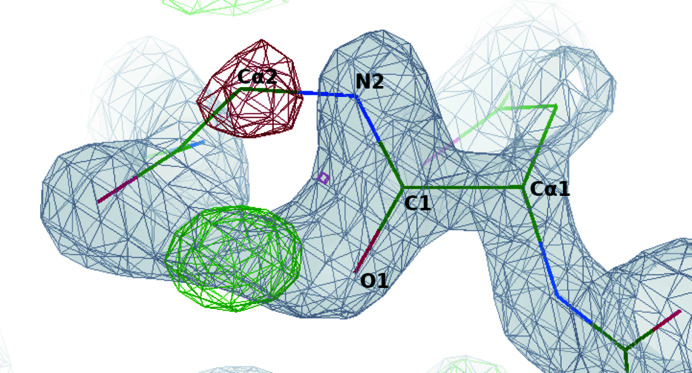
A reversed amide bond where negative difference density at the next C^α^ suggests an error in the previous residue. The example is a peptide bond between asparagine and glycine in a 1.86 Å resolution structure built by *Buccaneer* that was not used in this study. The 2*mF*_o_ − *DF*_c_ map is shown in grey. The positive and negative contours of the *mF*_o_ − *DF*_c_ map are shown in green and red, respectively.

The pepflip peak and next C^α^ difference density features in the main-chain neural network are quite unusual. They are not general validation metrics, but are designed to highlight specific errors that may occur during model building. The parameter minimization for the pepflip peak feature, as described in Section 2.3.8, resulted in a score of −3574, meaning there are at least 3574 residues (0.93%) with a pepflip peak and a target correctness of 0. Fig. 3 ▸ shows an example where it is useful to look at the density at the next residue. The amide oxygen and nitrogen need to swap positions, but both still fit the density well. However, the negative difference density at the next C^α^ suggests that there is something wrong with the previous residue.

**Figure 4:**
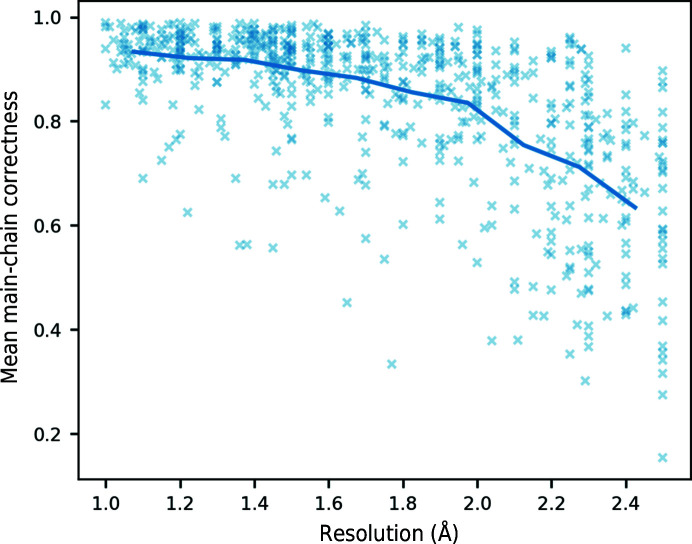
Resolution and mean main-chain target correctness for 639 structures in the training and test sets. The mean value for ten resolution bins is shown as a line.

Resolution is an interesting feature because it varies per structure and not per residue so, within a structure, it does not give any information about which residues are correct if used by itself. It was included to adjust the weights of other metrics; for example, at low resolution it is harder to distinguish side-chain positions and it is expected that rotamer score will be given more weight as uncommon conformations should only be built if the evidence for them is sufficient. However, the performance of *Buccaneer* is resolution-dependent. Fig. 4 ▸ shows that there is a higher proportion of incorrect residues at lower resolution, so the resolution feature will likely penalize the scores of residues in lower resolution structures. This is compensated for during automatic pruning by deleting residues with correctness values less than a fraction of the median value in the structure.

### 3.2. *Buccaneer* pipeline   

**Figure 5:**
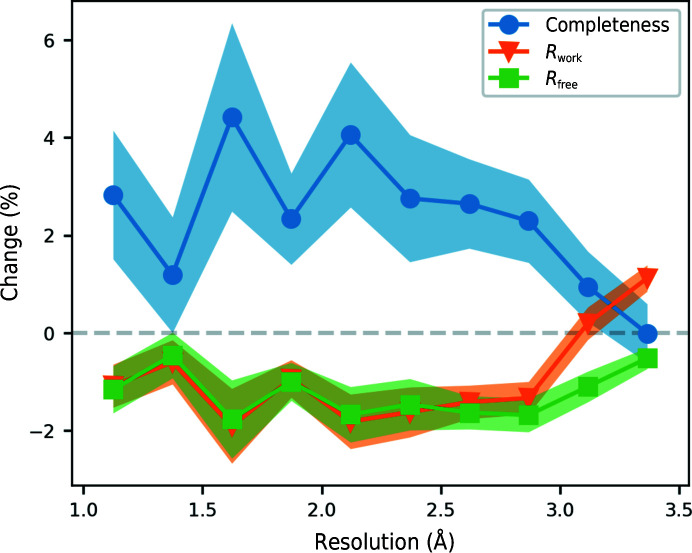
Change in completeness, *R*_work_ and *R*_free_ between the released pipeline and the chain-pruning pipeline. The 867 structures were divided into ten resolution bins and the mean and standard error of the change for each bin is shown.

Fig. 5 ▸ shows the change in completeness, *R*_work_ and *R*_free_ of the models produced by the *Buccaneer* pipeline on the addition of a chain-pruning step at the end of each iteration. Completeness is the percentage of residues in the refined deposited structure that have a matching residue in the model. Two residues were only considered to match if the N, C^α^ and C positions were all within 1 Å. At a resolution of 2.8 Å or better, the completeness improves by 2–3% and the *R* factors improve by 1–2%. Performance may be slightly less at very high resolution, but it is hard to tell owing to the noise in this region. At lower resolutions there is less improvement, but *R*_free_ still decreases. The gap between *R*_free_ and *R*_work_ widens at low resolution, which suggests that deleting some of the less correct chains is reducing the overfitting.

**Figure 6:**
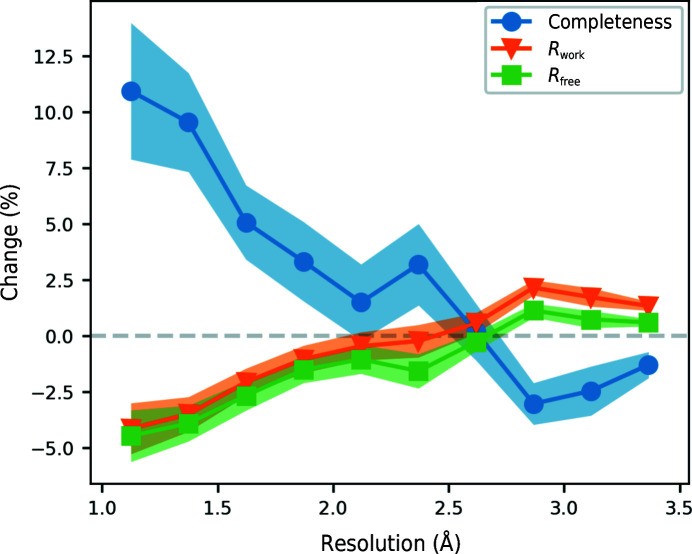
Change in completeness, *R*_work_ and *R*_free_ between the chain-pruning pipeline and the full pruning pipeline. The 867 structures were divided into ten resolution bins and the mean and standard error of the change for each bin is shown.

Fig. 6 ▸ shows the change in completeness, *R*_work_ and *R*_free_ of the pipeline models if an additional pruning step is added at the start of each iteration, other than the first, that prunes chains, residues and side chains. The effect of this change varies dramatically with resolution. The greatest improvement is seen at high resolution, where the completeness improves by around 10% and the *R* factors decrease by around 4% on average. The improvement quickly drops off at lower resolutions, with the full pruning step leading to worse pipeline performance below 2.6 Å resolution. Again, there is a difference between *R*_work_ and *R*_free_ that shows that pruning reduces overfitting.

**Figure 7:**
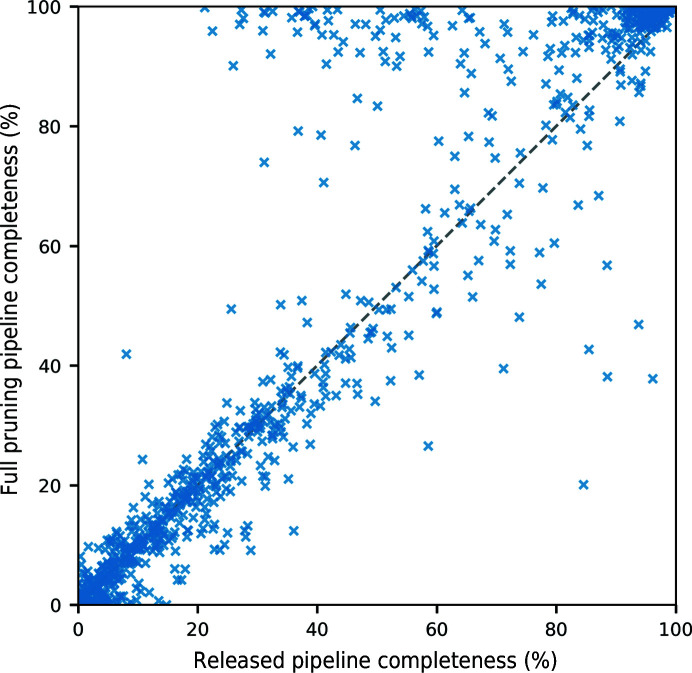
Completeness of the models from the released pipeline and the full pruning pipeline for the 867 structures tested.

Fig. 7 ▸ compares the completeness of the models from the released pipeline and the full pruning pipeline. There are 336 structures (39%) where the model from both pipelines had <20% completeness. Of these structures which performed badly in both pipeline versions, 173 (51%) were more complete in the released pipeline and 135 (40%) were more complete in the full pruning pipeline. At the other end of the scale, there are 183 structures where both pipelines produced a model with >80% completeness. Of these relatively complete structures, only 23 (13%) were more complete in the released pipeline, while 153 (84%) were more complete in the full pruning pipeline. There are also 63 structures (7%) at the top of Fig. 7 ▸ where the model from the full pruning pipeline has >90% completeness and the model from the released pipeline has <70% completeness, including an extreme example where the completeness increases from 21% to 100%.

**Figure 8:**
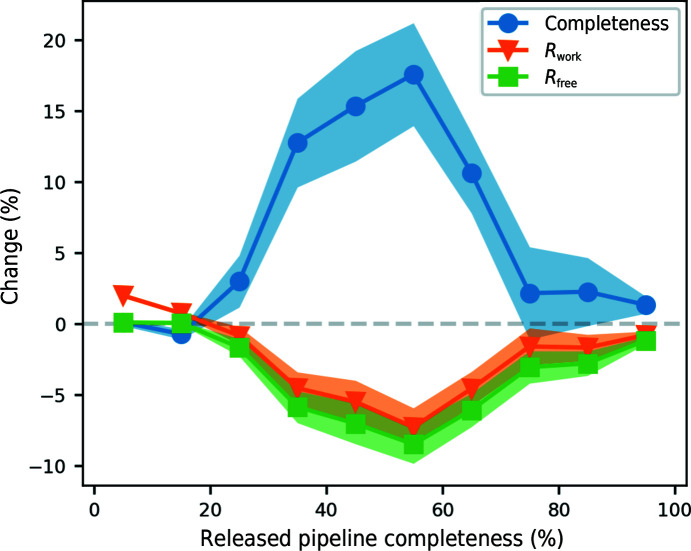
Change in completeness, *R*_work_ and *R*_free_ between the released pipeline and the full pruning pipeline against the completeness of the model from the released pruning pipeline. The 867 structures were divided into ten completeness bins and the mean and standard error of the change for each bin is shown.

An overview of the effect of the new pruning steps at different levels of completeness is shown in Fig. 8 ▸. For structures where the released pipeline produced models with around 50% completeness, the full pruning pipeline produced models with substantially higher completeness and lower *R*_free_ values on average. At higher levels of completeness there is much less room for improvement, but a small increase in the completeness and decrease in the *R* factors is still observed.

**Figure 9:**
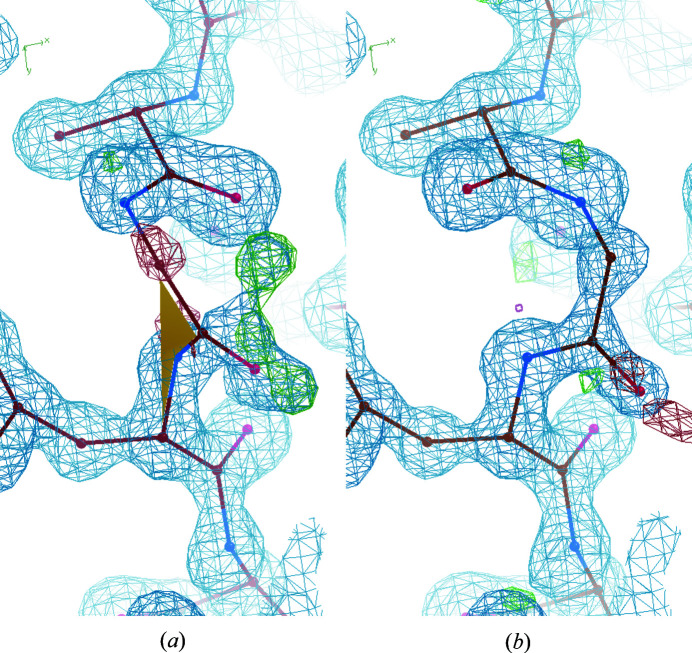
A section of PDB entry 4wn5 in (*a*) the model built by the released pipeline and (*b*) the model built by the full pruning pipeline. The 2*mF*_o_ − *DF*_c_ map is shown in blue. The positive and negative contours of the *mF*_o_ − *DF*_c_ map are shown in green and red, respectively. The yellow shaded area shows that the peptide bond is twisted, *i.e.* the ω angle is between 30° and 150°.

An example with high completeness in both pipeline versions is PDB entry 4wn5 (Fala *et al.*, 2015 ▸) at 1.15 Å resolution. The model produced by the released pipeline has a completeness of 90.14% and the model produced by the full pruning pipeline has a completeness of 98.59%. Much of the improvement in completeness is not owing to new parts of the structure being built, but because errors in the backbone conformations have been corrected. A section of both models is shown in Fig. 9 ▸. The peptide between alanine and glycine at the top of Fig. 9 ▸(*a*) is reversed, similar to the example shown in Fig. 3 ▸, so the glycine C^α^ atom is out of the density. The next peptide bond after glycine is also twisted, as indicated by the yellow shaded area. Both of these factors will contribute to a low correctness score. Deleting these residues allows *Buccaneer* to build the model correctly.

When using the predicted correctness scores for pruning, a decision needs to be made about the threshold used for selection. Because the scores cannot predict correctness with 100% accuracy, any chosen threshold will prune some correct residues and leave some incorrect residues. The thresholds tested were 0.2 times the median for whole chains and 0.5 times the median for residues and side chains. Other thresholds have not yet been tested, but the optimum value is likely to depend on the stage of model building. More caution needs to be taken at the end of the pipeline because it is usually easier for the user to fix an incorrect conformation than to build a missing feature. If pruning is performed during the pipeline, before further cycles of *Buccaneer*, then it can be less cautious because correct residues that are mistakenly deleted should be automatically rebuilt. However, a balance is still required because deleting more correct residues than incorrect residues can reduce the quality of the phases and make building more challenging.

## 4. Conclusion   

The correctness of 382 485 residues in 639 *Buccaneer* models was assigned by automatic comparison with the models deposited in the PDB for these structures. Residues were given correctness values of either 0 or 1, which was performed separately for main chain and side chains. This method of producing target correctness values is not perfect, but the vast majority of residues will be labelled correctly. Manual labelling of each residue is too slow and it is important to have a large number of data points for the machine learning to work well.

Regression was carried out for 511 of the structures using two neural networks to predict the correctness by combining many features of each residue. The input features include map-to-model correlation, density values, *B* factors, clashes, Ramachandran scores, rotamer scores and resolution. Using regression instead of classification means that intermediate correctness scores can be obtained, hopefully for residues where it is not obvious whether the conformation is correct or not. If scores of less than 0.5 are classed as incorrect, the trained networks correctly categorize 92.3% of main-chain atoms and 87.6% of side chains in the set of 128 structures that were not used for training. The correctness predictions show no sign of overfitting, but they are expected to work best on structures similar to those used in the training set, *i.e.* mostly complete structures with resolutions better than 2.5 Å.

A *Coot* ML Correctness script was written to calculate the predicted correctness values and show them to the user as a validation tool. This helps to quickly identify the worst parts of a structure for further examination. The aim is not to have high correctness scores for the whole structure as, owing to the reliance on *Z*-scores in the input features, the score is relative to the whole structure. Deleting poor parts of the structure will decrease the correctness scores for the remaining model. The script also contains an automatic pruning function for deleting whole chains, residues and side chains with low correctness scores. It can be called with default parameters from the *Coot* graphical user interface or with custom parameters via the scripting interface.

The pruning function was incorporated into the *Buccaneer* pipeline in *CCP*4*i*2 to prune whole chains at the end of each cycle and also individual residues and side chains at the beginning of each cycle. The pipeline changes were tested on 867 structures at 1–3.5 Å resolution. The final pruning of whole chains leads to improved models and the improvement is not very dependent on resolution. In contrast, the initial pruning of residues and side chains gives large improvements at high resolution but often leads to worse models at low resolution. Hence, it is only recommended to include residue-level pruning when the resolution is better than 2.6 Å. There are many structures that have changed from being partially built to almost fully built with the addition of the new pruning steps.

## 5. Future work   

Although the addition of the pruning step leads to improvements in the *Buccaneer* pipeline, the correctness score is far from optimal. One of the main problems is that machine learning was carried out as a mixture of classification and regression. Regression was used in order to obtain a continuous correctness score instead of a binary classification. However, as the target data were categorical, *i.e.* all samples had a target correctness of 1 or 0, it would have been better to use a classifier and obtain continuous values in the form of the predicted probabilities for each class. Another option would be to perform regression against a different, continuous target; for example, the r.m.s.d. between the atoms of the query structure and the reference structure. This has the advantage that no cutoff has to be chosen, although it may also have difficulties in that a residue built into the solvent 5 Å away from the structure is no different to one 10 Å away. Classification using the r.m.s.d. could be a solution to this, but it does not have to be binary: for example, the classes could be an r.m.s.d. of <0.5 Å, <1 Å, <2 Å and ≥2 Å.

After choosing the training target and either classification or regression, the model should be examined in more detail. For this study a neural network model was used, and hyperparameters such as the learning rate and the regularization term were kept at their default values. However, other models such as a decision tree or a random forest should also be explored as they may produce better results, and hyperparameters should be tuned for optimum performance.

The structures built by *Buccaneer* in the easy reduced set contain mostly correct residues and side chains. This imbalance means that the networks will be better at identifying correct residues than incorrect residues and explains the high false-positive rate. Incorrect residues are identified, but these are likely to be obvious errors such as residues built into the solvent. Resampling should be considered to either undersample the correct residues or oversample the incorrect residues. More difficult cases could be included, but these need to be chosen carefully. The models built by *Buccaneer* are often either largely correct or composed of small fragments built into noise, and the incorrect residues in these two extremes will have very different features. The correctness score was not intended to help in the latter case, where better initial phases may be required.

As mentioned in Section 4, owing to the use of *Z*-scores in the features, the correctness of a residue is not only dependent on its immediate environment but on the whole structure. This is counterintuitive and should be changed. Map values will still be needed in the features, but dependencies on the absolute scale of the map or the solvent content of the structure may be introduced depending on how they are measured.

It would also be beneficial to have a correctness score using features that can be calculated quickly for an individual residue for the purpose of providing feedback during model building. This could be provided in addition to a more accurate score that is only calculated after refinement. For the quick score, difference-map values should not be used as they would need to be recalculated after the model changes. It may also be necessary to remove *B* factors from the features unless they can be obtained quickly, for example using shift-field refinement (Cowtan & Agirre, 2018 ▸). Other features that are missing from the current implementation should be investigated. It is likely that more generic geometric scores would be helpful, such as the χ^2^ values of the bond and angle restraints displayed in *Coot* after real-space refinement.

## 6. Availability   

The *Coot* ML Correctness script and scripts used for training the neural networks are available at https://doi.org/10.15124/44145f0a-5d82-4604-9494-7cf71190bd82. *Coot *version 0.8.9.2 or later is required for the script to work. The new pruning steps added to the *Buccaneer* pipeline in *CCP*4*i*2 will be available in *CCP*4 version 7.1. They can be turned on and off from the Options tab on the Input page of the task.

## Supplementary Material

Automatic creation of molecular-replacement test sets. DOI: 10.1107/S2059798320009080/qj5003sup1.pdf

Data sets and scripts.: https://dx.doi.org/10.15124/44145f0a-5d82-4604-9494-7cf71190bd82

## Figures and Tables

**Table 1:** Summary of the features used to predict main-chain and side-chain correctness

Features	Main/side chain	Section
Map-to-model correlation	Both	2.3.1
Mean best density *Z*-score	Both	2.3.2
Minimum best density *Z*-score	Both	2.3.2
Minimum difference density *Z*-score	Both	2.3.2
Maximum *B*-factor *Z*-score	Both	2.3.3
Maximum *B*-factor change *Z*-score	Both	2.3.3
Maximum atom overlap	Both	2.3.4
Resolution	Both	2.3.5
Ramachandran score	Main	2.3.6
Maximum peptide twist	Main	2.3.7
Pepflip peak	Main	2.3.8
Difference density *Z*-score at the next C^α^	Main	2.3.2
Rotamer score	Side	2.3.9

**Table 2:** Summary of the *CCP*4*i*2 *Buccaneer* pipeline versions that were tested

Pipeline	Initial full pruning	Final chain pruning
Released (*CCP*4 7.0.076)	No	No
Chain pruning	No	Yes
Full pruning	Yes	Yes

**Table 3:** Trained neural network COD for the training and test sets Values are the mean COD after training with 100 different random-number seeds with one standard error in parentheses.

Network	Training-set COD	Test-set COD
Main chain	0.6534 (2)	0.6665 (2)
Side chain	0.6004 (2)	0.6073 (2)

**Table 4:** Quality metrics for the main-chain and side-chain neural networks on the residues in the test set, assuming that residues with correctness scores of ≥0.5 are predicted to be correct Equations for these metrics are given in (3)–(10).

Network	Main chain	Side chain
Accuracy (%)	92	88
Error (%)	8	12
Sensitivity (%)	97	92
Specificity (%)	77	79
False-negative rate (%)	3	8
False-positive rate (%)	23	21
Precision (%)	94	90
*F*_1_ score (%)	95	91

**Table 5:** Test-set COD for the main-chain neural network after it has been trained with individual features removed Values are the mean COD after training with 100 different random-number seeds with one standard error in parentheses.

Missing main-chain feature	Test-set COD	Decrease
No missing feature	0.6665 (2)	0.0000
Pepflip peak	0.6646 (3)	0.0019
Maximum *B*-factor *Z*-score	0.6642 (2)	0.0023
Difference density *Z*-score at the next C^α^	0.6624 (2)	0.0041
Maximum *B*-factor change *Z*-score	0.6621 (2)	0.0044
Minimum best density *Z*-score	0.6613 (2)	0.0052
Maximum peptide twist	0.6604 (3)	0.0061
Minimum difference density Z-score	0.6598 (2)	0.0067
Maximum atom overlap	0.6592 (2)	0.0073
Mean best density *Z*-score	0.6570 (3)	0.0095
Ramachandran score	0.6563 (2)	0.0102
Resolution	0.6377 (3)	0.0288
Map-to-model correlation	0.6087 (3)	0.0578

**Table 6:** Test-set COD for the side-chain neural network after it has been trained with individual features removed Values are the mean COD after training with 100 different random-number seeds with one standard error in parentheses.

Missing side-chain feature	Test-set COD	Decrease
No missing feature	0.6073 (2)	0.0000
Minimum difference density *Z*-score	0.6038 (2)	0.0035
Maximum atom overlap	0.6027 (2)	0.0046
Maximum *B*-factor change *Z*-score	0.6021 (2)	0.0052
Minimum best density *Z*-score	0.6000 (2)	0.0073
Mean best density *Z*-score	0.5968 (2)	0.0105
Maximum *B*-factor *Z*-score	0.5901 (2)	0.0172
Resolution	0.5874 (2)	0.0199
Rotamer score	0.5835 (2)	0.0238
Map-to-model correlation	0.5566 (2)	0.0507
