# Supplementary material for: Predicting protein model correctness in Coot using machine learning
Source: Acta Crystallogr D Struct Biol. 2020 Jul 27;76(Pt 8):713–23. doi: 10.1107/S2059798320009080 (PMC7397494; doi:10.1107/S2059798320009080)
Supplement: Supplementary file 1 [file d-76-00713-sup1.pdf]

Automatic Creation of Molecular Replacement Test Sets

Paul S. Bond, Keith S. Wilson and Kevin D. Cowtan

Abstract

When testing software, it is important to use a large number of test cases so that the significance of performance improvements can be assessed. Molecular replacement (MR) is the most commonly used structure solution technique for X-ray diffraction data, and this has led to the development of a number of automated MR solution pipelines. Since different software tools often focus on different problem classes, for example data resolution, it is useful to be able to generate a custom test set for a given task. A program was written for the automatic creation of MR test sets to address this problem. In addition to this, a large example test set was prepared using the program. Two thousand structures, evenly spread between 1 Å and 3.5 Å resolution, were chosen at random from the PDB. The structures had to meet quality thresholds, measured using wwPDB validation percentiles, and not contain any chains with 50% or more sequence identity to chains in other chosen structures. After checking the structure factor data and refining the deposited structure, 1800 of the structures were deemed suitable. A search for structural homologues was carried out for the 2100 unique chains in these structures and 15532 MR models were made from the homologues, 11183 of which led to a refined solution. Two reduced test sets were produced with only one MR model per structure. The full reduced test set contains 1351 structures for the full resolution range and a wide range of initial phase qualities. The easy reduced test set contains 639 structures with better than 2.5 Å resolution, all with good quality phases. The BUCCANEER model building pipeline from CCP4i built models with a mean completeness of 39% for the full set and 87% for the easy set. The test set creation program and the example test sets are available as a resource for the community.

1 Introduction

Large test sets are required to determine whether new program developments lead to statistically significant performance improvements. It is likely that a change will lead to better performance in some cases and worse performance in others, so it is not sufficient to test only one structure and assume that the same improvement will be seen across the board. Testing more independent structures will lead to a smaller standard error in the mean improvement. There is often a trade off between the speed and accuracy of a program, and being able to reliably measure differences means a more informed decision can be made about whether a change should be implemented.

The best way to proceed with structure solution in X-ray crystallography depends on the information currently available. A high resolution structure with only a single helix

placed and a low resolution structure that requires some large scale domain movements present very different problems that need different methods to solve. As an example, ACORN [1] is a very powerful phase refinement procedure but it is only applicable to high resolution data. Having a large test set with a range of resolutions and initial phase qualities makes it possible to predict where the program would be beneficial, allowing for expert pipelines to be created that use different approaches depending on factors such as resolution.

Most program developers will already have their own test sets but these are usually not publicly available. For instance, the automated model building program ARP/wARP [2, 3] is available via a web service and the data submitted to that can be used to train ARP/wARP algorithms. The user may also give permission for their data to be shared with a wider audience, in which case it may be available on request. However, without further curation, user submitted data may not form a uniform or representative sample and so may not be suitable for any specific research question.

The work presented here has two main aims. Firstly, to create a large, publicly available test set with structures that are evenly spread across a range of resolutions and phase qualities. The independence of individual tests is controlled by ensuring the target structures do not have high sequence homology to each other. The second aim is to release the program used to make the test set so it can be repeated to create new test sets with varied parameters, such as a set containing only low resolution structures.

A source of initial phases is needed to test model building programs and phase refinement programs. If the structure contains heavy atoms, and anomalous data has been deposited at suitable wavelengths, then SAD/MAD phasing could be used. However, it is a minority of structures in the PDB [4] that meet these requirements. It was decided to use molecular replacement (MR) as a source of initial phases because only mean structure factor amplitudes are needed and an MR model can be created from any homologous structure. A range of test cases can be produced by varying the similarity of the homologues, as well as the fraction of the target structure that they represent. This also means that the resulting test sets could be used to assess the performance of molecular replacement programs.

2 Methods

Calculations were performed on a Scientific Linux 7.7 server with two AMD EPYC 7451 CPUs and 256 GB RAM. Programs were sourced from CCP4 7.0.076 [5].

2.1 Test Set Creation

2.1.1 Choosing Target Structures

A list of all deposited chains was downloaded from the RCSB PDB [4] and filtered to contain only L-polypeptide chains of at least 20 residues from structures solved by X-ray diffraction. In order to ensure an even spread across a range of resolutions, structures were placed into 10 bins between 1 Å and 3.5 Å resolution. Two hundred structures were chosen at random from each bin to give 2000 targets in total. Structures were only chosen if they were of suitable quality. Five statistics from the PDB validation report were used as overall quality indicators: R-free, calculated by DCC [6]; clashscore, Ramachandran outliers and sidechain outliers, calculated by MolProbity [7]; and real-space R-value Z-score (RSRZ) outliers, calculated by EDS [8]. R-free had to be in at least the 50th percentile relative to similar resolution structures and the other statistics had to be in at least the 40th percentile.

To stop common protein families being represented multiple times, structures were also rejected if they contained a chain with $\geq 50\%$ sequence identity to a chain in an already chosen structure. This was assessed using cluster numbers available from the PDB, which are pre-calculated using BLASTCLUST [9] at various sequence identity thresholds. Resolution bins with fewer structures were considered first to avoid running out of qualifying structures.

Other than needing to contain a protein chain with at least 20 residues, there were no restrictions on the content or size of the structures. Structures can be hetero-multimers and may contain other entities that make model building more complicated, such as nucleic acids, cofactors and glycosylation.

2.1.2 Preparing Structure Data

A FASTA format sequence file was downloaded from the RCSB PDB with entries for all 2000 structures. For each structure, sequences of protein chains with at least 20 residues were extracted and written to a separate sequence file. A second sequence file was written containing only the unique sequences as some programs require a file without duplicate entries.

Reflection data were converted from CIF format to MTZ format using CIF2MTZ. The files may contain amplitudes or intensities and data for Friedel pairs may be combined or held separately for anomalous phasing. In order to make the files contain the same type of structure factor data, they were all processed with CTRUNCATE [10], which converts intensities to amplitudes, anomalous data to mean data and also performs anisotropy correction. A new free-R flag was then assigned using the CCP4 utility FREERFLAG and column labels were standardised.

Unknown ligand (UNL) residues were removed from the deposited coordinates, which

were then refined with REFMAC [11, 12] for 10 cycles. By default, the program exits if it encounters a new ligand, but this behaviour was altered to avoid too many failures. Instead, REFMAC was told to proceed with refinement using a dictionary description it creates from the ligand coordinates. The final R-factor was compared to the R-work reported in the PDB and the structure was rejected if it was more than 5% higher. Structures were also rejected if the overall data completeness was less than 90%.

2.1.3 Choosing Homologues

For each unique chain in the selected structures, a search for structural homologues was performed using GESAMT [13] on a local copy of the PDB, updated on the 31st July 2019, containing 153578 structures. This search is very time consuming, but was sped up considerably by using a pre-constructed GESAMT archive and searching in parallel over 96 threads. The GESAMT archive consists of compressed binary files containing only protein Cα coordinates that can be read very efficiently [14].

Homologues with $\geq$ 70% sequence identity were removed from the results as these are too similar to make challenging MR models. At the other end of the scale, the search results also contain large numbers of chains that are too distant to be suitable for MR, so only chains with an RMSD less than 3 Å and Q-score more than 0.2 were considered. For each target chain, up to 10 homologues were chosen from the filtered list in order of descending Q-score. If a homologue had a sequence identity more than 70% or an RMSD less than 1.5 Å to a previously chosen homologue then it was eliminated.

2.1.4 Preparing MR Models

Each homologue chain was superposed onto its target chain using GESAMT to produce a sequence alignment. The sequence files from GESAMT were converted to CLUSTAL [15] format alignment files for SCULPTOR [16], which was used for preparing the MR models. Alternate conformations were removed from the input model and default parameters were used for pruning.

2.1.5 Molecular Replacement

Molecular replacement was carried out for each SCULPTOR model using PHASER [17]. This was done for each model individually, so there will be no solutions containing multiple components other than multiple copies of the same model. In a real MR scenario the target structure is not known, so only sequence identity from the GESAMT alignment was given to PHASER, which then made its own estimate of the model RMS error. The composition of the asymmetric unit was defined using the counts of each atom type in the deposited coordinates. The number of copies to search for was also known from the deposited structure. In order to speed up cases that lacked a clearly significant

solution, the solution list was purged to keep only the top solution at the rotation function, translation function and refinement stages.

The placed MR models were refined for 10 cycles with REFMAC using default parameters. Phases from the refined MR model were compared to phases from the refined deposited structure using the CCP4 utility CPHASEMATCH, which will correct for alternate origins chosen during molecular replacement.

2.2 Test Set Reduction

The full test set contains multiple placed MR models for each structure, up to 10 individual models for each unique chain. This could be useful to see whether a model building program can produce a correct structure from a variety of starting points, but it is not optimal in many circumstances. It takes much longer to run the whole test set and results obtained for separate models are less independent. To address these issues, two smaller test sets were constructed by choosing a single model for each target structure.

The first, named the full reduced test set, aims to have a broad range of phase qualities. Phase quality was measured using F-map correlation, which is the correlation coefficient between the structure factor amplitudes of the map from the refined MR model and the map from the refined deposited model, weighted by the cosine of the phase difference. Seven F-map correlation bins were created between 0.2 and 0.9. The models for each structure were checked in a random order to see if they belong in the bin with the least number of structures. If no model was found, then the bin with the next least structures was considered until a suitable model was found.

The second is named the easy reduced test set for cases that should be easily solved by automatic model building. It was created using the same method as the full reduced test set, but only with structures where the resolution is 2.5 Å or better and using 5 F-map correlation bins between 0.7 and 0.95.

2.3 Model Building with BUCCANEER

Automated model building was carried out on both reduced test sets using the BUCCANEER pipeline from CCP4i [18, 19] with default options for molecular replacement, including model seeding, which adds every third residue in the MR model to the input model. In some cases the target structure was a selenomethionine derivative and MSE residues were built instead of MET. The final models from the BUCCANEER pipeline were superposed onto the refined deposited structure using CSYMMATCH, which searches for the best fit using symmetry operations and allowed origin shifts.

Table 1: Reasons for 200 out of the 2000 structures being rejected.

Count	Reason
114	Data completeness below 90%
45	Error during refinement
34	Refined R-work more than 5% higher than reported
4	Error in the deposited coordinate file
1	No symmetry information in the structure factor data
1	No standard deviations in the structure factor data
1	Error during the least squares fit when converting amplitudes

3 Results and Discussion

3.1 Test Set Creation

The initial structure selection can be altered by changing the minimum and maximum resolution, number of resolution bins, number of structures per resolution bin, maximum sequence identity, and validation thresholds. The maximum sequence identity may need to be increased and the validation thresholds decreased if looking for large numbers of structures at high or low resolution where there are less structures available.

When choosing the number of structures, it should be taken into account that not all will be suitable. Out of the 2000 structures initially selected, only 1670 (83.5%) had one or more refined MR models, which may be correctly or incorrectly placed. 200 structures were rejected at some stage during data preparation, reasons for which are shown in Table 1.

The most common reason for rejection was low data completeness. It was decided that a threshold of 90%, rejecting 5.7% of structures, was acceptable but this can be changed by the user. The next most common reason was errors occurring during refinement, which were mainly due to ligand atoms being absent in the library. The deposited structures had to refine to within 5% of the reported R-work using default parameters in REFMAC. If the structure was originally refined using non-default procedures, such as twinned refinement or anisotropic B-factor refinement, then it will have a much higher chance of being rejected at this stage.

Resolution and refined R-free for the 1800 structures that passed are shown in Figure 1. As expected, lower resolution structures generally have higher R-factors. There were structures with much higher R-free values but these were rejected.

The 1800 chosen structures had 2100 unique chains between them with 15551 structural homologues chosen from the GESAMT archive searches. The maximum number of homologues to choose for each target chain can be modified, along with how similar the homologues can be to the target and how similar they can be to each other. Searching

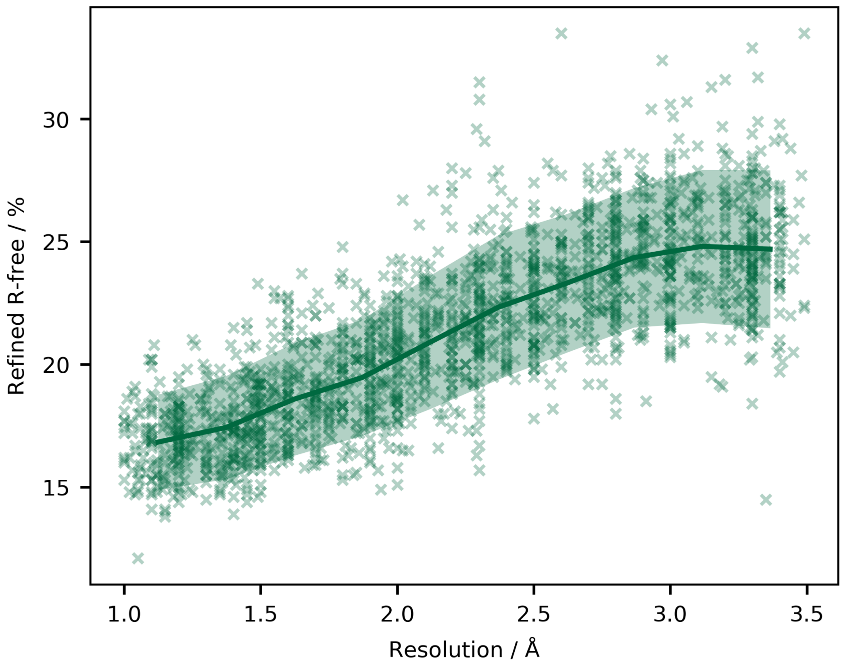

Figure 1: Resolution and refined R-free for the 1800 structures that passed the data preparation stage. Raw data are shown as crosses. Mean values for 10 resolution bins are shown as a solid line. The shaded area shows one standard deviation above and below the mean.

for structural homologues instead of sequence homologues should lead to a lower failure rate during molecular replacement. In a real molecular replacement scenario this is obviously not possible because the target structure is not known.

SCULPTOR made an MR model for 15532 of the homologues. In the other 19 cases an empty coordinate file was produced. PHASER rejected the input for 8 of the models. In one case this was due to a poor ensemble model from a homologous structure containing two models that did not correlate well with each other. The other 7 errors were from two structures that had some reflections with negative structure factor amplitudes. PHASER failed to find a solution for 4341 out of the 15524 runs that terminated successfully, leaving 11183 placed MR models that were refined.

The GESAMT Q-score is a measure of alignment quality that takes into account both RMSD and the length of the alignment [13]. It increases from 0 to 1 as the structural similarity of the two structures increases, so models with higher Q-scores should be more successful during molecular replacement. PHASER provided a Log Likelihood Gain (LLG) and an estimated RMSD for 10896 of the models it placed. Figure 2 shows GESAMT Q-score and F-map correlation for these models.

The GESAMT Q-scores in Figure 2 are from the superposition step and not the structural homologue search. There are occasional differences between these values, hence a few homologues have Q-scores below 0.2 despite that being the minimum during selection. More than half (56%) of the placed MR models have F-map correlations below 0.15. It

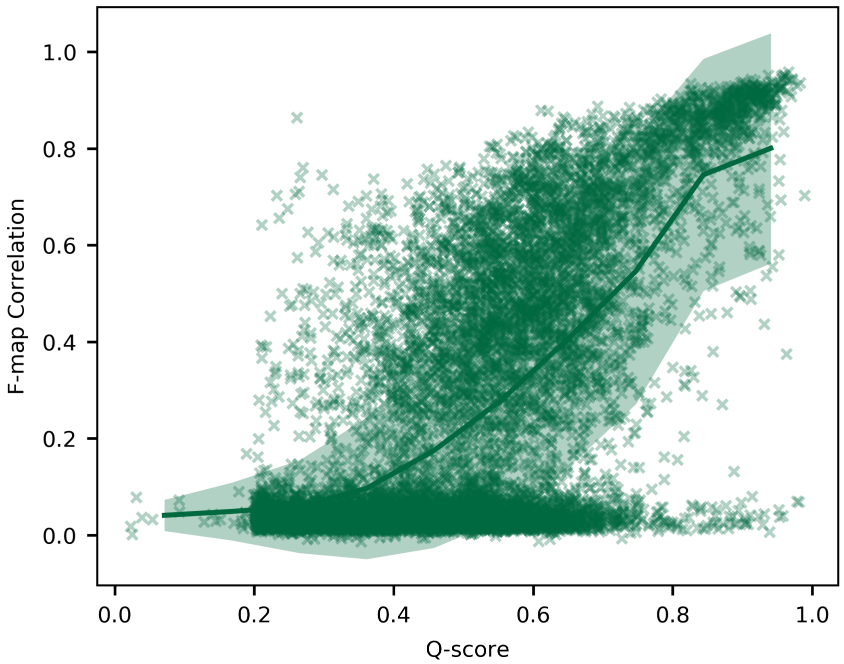

Figure 2: GESAMT Q-score and F-map correlation for 10896 models placed with PHASER and refined using REFMAC. Mean and standard deviation for 10 Q-score bins are overlaid.

is likely that this cluster is formed mostly of incorrect solutions.

Increasing the minimum Q-score between the homologue and target chains should increase the success rate of molecular replacement. As expected, Figure 2 shows a positive correlation between Q-score and F-map correlation, but the structural similarity of the homologue is not the only factor to take into account. One model has a Q-score of 0.94 but an F-map correlation of only 0.01. The target structure is 6HV7 at 3.4 Å resolution, which has 6602 residues comprising two copies of 14 unique chains. Both the target, chain I, and the homologue, chain J of 4R3O, have 204 residues built. The GESAMT superposition aligns 203 residues with an RMSD of 0.70 Å and a sequence identity of 54%. Although the model is very similar, it is small in comparison to the full structure and the resolution is low so PHASER could not produce a correct solution.

In another example, a model has a Q-score of 0.26 but this leads to an F-map correlation of 0.86. The target structure is 5MN7 at 3.3 Å resolution. It has two copies of a 305 residue chain with 303 residues in the deposited model. The homologue is chain A from 2Q1Y with 305 residues, but the GESAMT superposition only aligns 160 residues with an RMSD of 0.75 Å and a sequence identity of 64%. The Q-score is low because it is calculated for just over half of the full length. However, the sequence alignment produced is for the full length and SCULPTOR is still able to produce a good model despite the alignment containing an incorrect gap.

Not all of the models with low F-map correlations are incorrect. Correctly placed solutions can still lead to low F-map correlations if the model is dissimilar or makes up a small

Table 2: Percentage of correct solutions for different LLG ranges. Solutions with F-map correlations greater than 0.15 are classed as correct.

Min LLG	Max LLG	Number of Solutions	Correct / %
0	20	718	1.4
20	40	3784	8.4
40	60	1795	29.4
60	80	822	50.1
80	100	474	70.3
100	120	346	80.6
120	140	259	92.3
140	160	211	93.4

fraction of the complete structure. In these cases model building will be challenging but it might be possible to improve the phases using density modification or further molecular replacement with other parts of the structure. Log Likelihood Gain (LLG) is often used to judge the correctness of a solution. Table 2 shows the percentage of correct solutions for different LLG ranges, assuming that solutions with F-map correlations greater than 0.15 are correct.

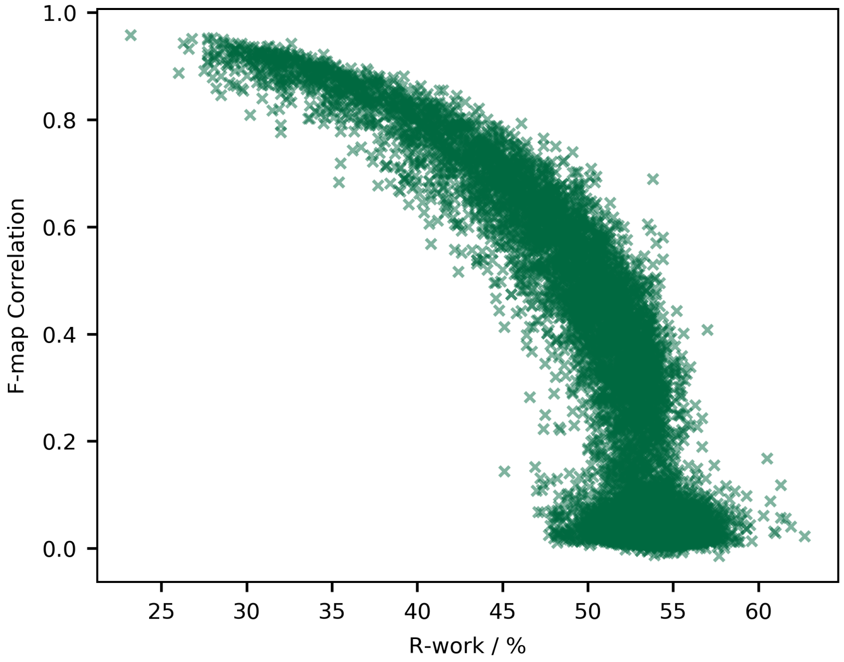

Figure 3: R-work and F-map correlation for 10896 models placed with PHASER and refined using REFMAC.

When determining whether a solution is correct, it is best to look at LLG in combination with other statistics, such as the refined Translation Function Z-score (TFZ) and the number of packing clashes. The R-factors of the refined MR model are also useful. Figure 3 shows how F-map correlation varies with R-work. Solutions with F-map correlations

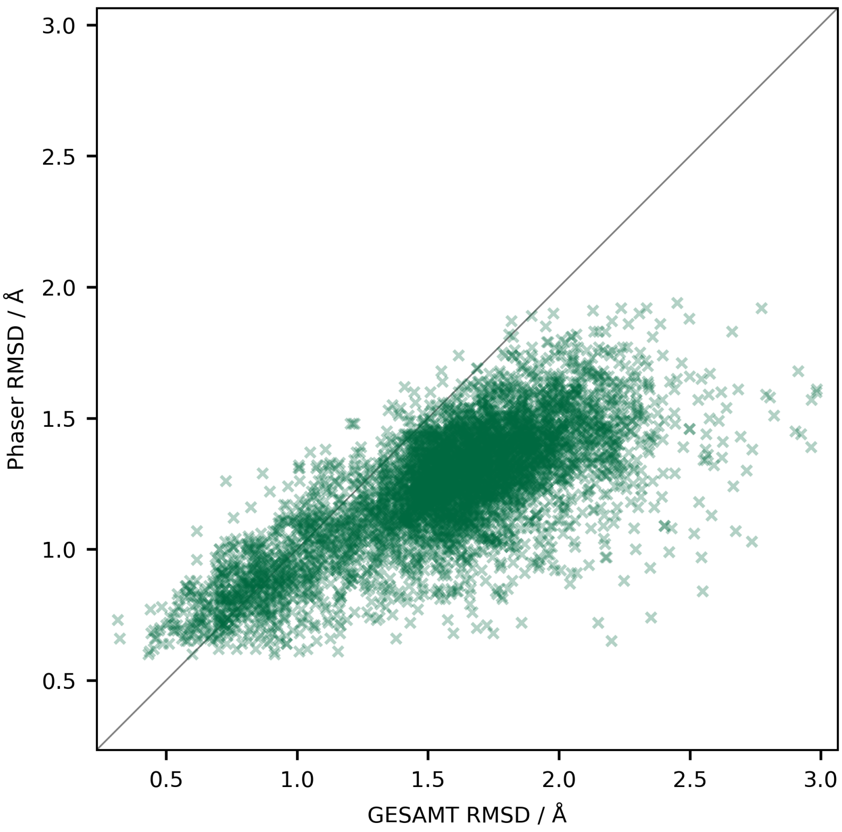

Figure 4: GESAMT RMSD vs PHASER RMSD for 4773 models with F-map correlation greater than 0.15.

less than 0.15 generally have R-work values above 47%. However, most of the correct solutions also have R-factors in this region. A refined R-work of 50–55% does not give much information about the correctness of the solution, but values less than 45% are very likely to be correct solutions.

PHASER produces an estimate of the RMSD between the model and the true structure. This can be compared to the RMSD from GESAMT, which is calculated for the aligned region of the homologue and target chains. Figure 4 shows RMSD from both PHASER and GESAMT. There is a positive correlation but PHASER overestimates low RMSD values and underestimates high RMSD values compared to the actual values from GESAMT.

The purpose of performing molecular replacement was to get some placed models with realistic errors for testing different model building strategies. A less realistic route would have been to superpose the MR model onto all the copies of the target chain using GESAMT. If the goal is to create a test set for assessing molecular replacement then the creation script can be stopped after SCULPTOR creates the models.

Because the aim was not to solve as many cases as possible, neither PHASER nor REFMAC were used to their full potential. Intensities, if originally available, were converted to amplitudes despite intensities being preferred for the LLGI function [20], as it was de-

Table 3: Overall performance of the CCP4i BUCCANEER pipeline on both the full and easy reduced test sets. Values shown are the mean ± one standard error.

	Full Set (1351 structures)	Easy Set (639 structures)
Completeness / %	39.5 ± 0.8	86.7 ± 0.5
R-work / %	43.1 ± 0.2	30.6 ± 0.2
R-free / %	48.9 ± 0.2	34.5 ± 0.3

cided that having standardised data provided a more useful comparison. Purging all but the top solution also severely limited the performance of PHASER, but had to be done to save time when running thousands of jobs without clear solutions. It is usually preferable to first refine MR models in REFMAC using rigid body refinement or to include jelly body restraints for many cycles, especially when there are large scale differences between the model and the true structure, but this was also not done in order to save time.

3.2 Test Set Reduction

There are 1351 structures in the full reduced test set with resolutions between 1.0 and 3.5 Å and F-map correlations between 0.2 and 0.9. The easy reduced test set has 639 structures with resolutions between 1.0 and 2.5 Å and F-map correlations between 0.7 and 0.95. In both test sets, cases are spread evenly across the resolution and F-map correlation ranges.

3.3 Model Building with BUCCANEER

The overall performance of the CCP4i BUCCANEER pipeline on the reduced test sets is shown in Table 3. Completeness is the percentage of residues in the refined deposited structure that have a matching residue in the model. Two residues were only considered matching if the N, CA and C positions were all within 1 Å. As expected, performance is much better on the easy reduced test set. Performance on some structures will be limited due to the presence of non-protein components, such as nucleic acids, that BUCCANEER is not able to build.

Figure 5 shows how completeness varies with resolution for 389 cases with a starting F-map correlation of 0.7 or more. There is more of a drop in performance at low resolution than was observed for simulated low resolution experimentally phased datasets, which still had a mean completeness higher than 50% at 3.4 Å resolution [21]. There are many factors contributing to this difference. Firstly, the simulated datasets have better phase information than would normally be obtained at low resolution. The maps produced by experimental phasing and molecular replacement are also quite different. Even at a similar level of F-map correlation, an experimentally phased map will likely have more uniformly distributed errors. Molecular replacement maps contain model bias that makes

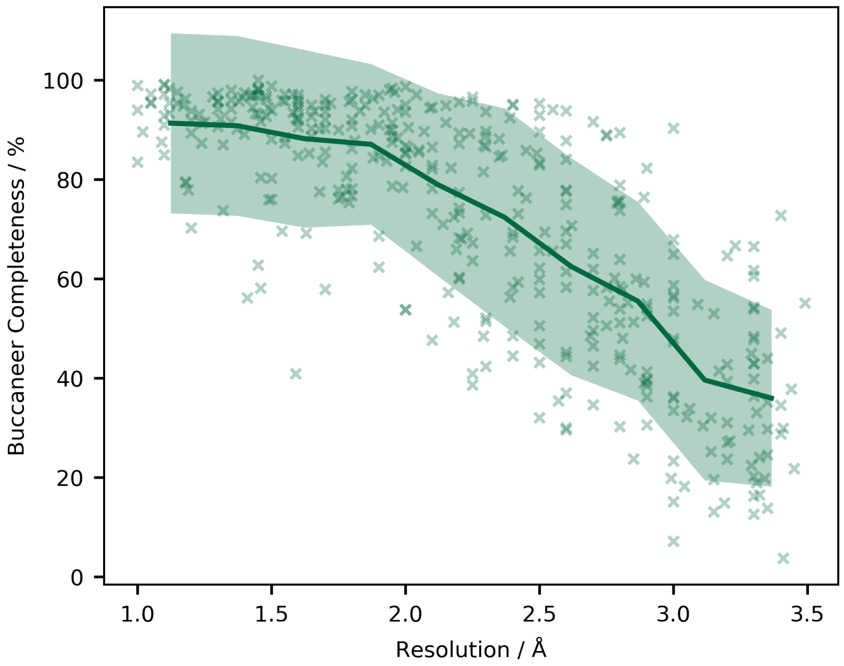

Figure 5: Resolution and completeness of the BUCCANEER model for 389 structures in the full reduced test set with F-map correlation 0.7 or more. Mean and standard deviation for 10 resolution bins are overlaid.

model building more challenging. BUCCANEER also uses different options depending on the initial source of phases. If an MR model is available it will be used for Cα seeding. If experimental phases are provided they will be given to REFMAC for MLHL refinement. Lastly, the completeness metric used in this study is slightly stricter as it matches residues using N and C positions as well as Cα positions. Both metrics used a tolerance of 1 Å for correct atomic positions. A drop in performance at low resolution is expected due to this as two similar quality models are less likely to be within a fixed tolerance of each other.

Figure 6 shows how completeness varies with phase quality for 911 cases with resolutions 2.5 Å or better. BUCCANEER is known to be sensitive to phase quality [18]. Below an F-map correlation of 0.4 there is only one model with more than 50% completeness. However, all of the cases contain some correct phase information, from which it would hopefully be possible to bootstrap a correct solution.

4 Conclusion

A program has been developed for the automatic creation of molecular replacement test sets. It starts by choosing good quality target structures with diverse protein sequences that span a range of resolutions. A search for structural homologues is then carried out and molecular replacement models are prepared from the homologues. The program can be terminated at this point, or it can continue to perform molecular replacement and refine the placed solutions when a source of initial phases is needed. Many parameters

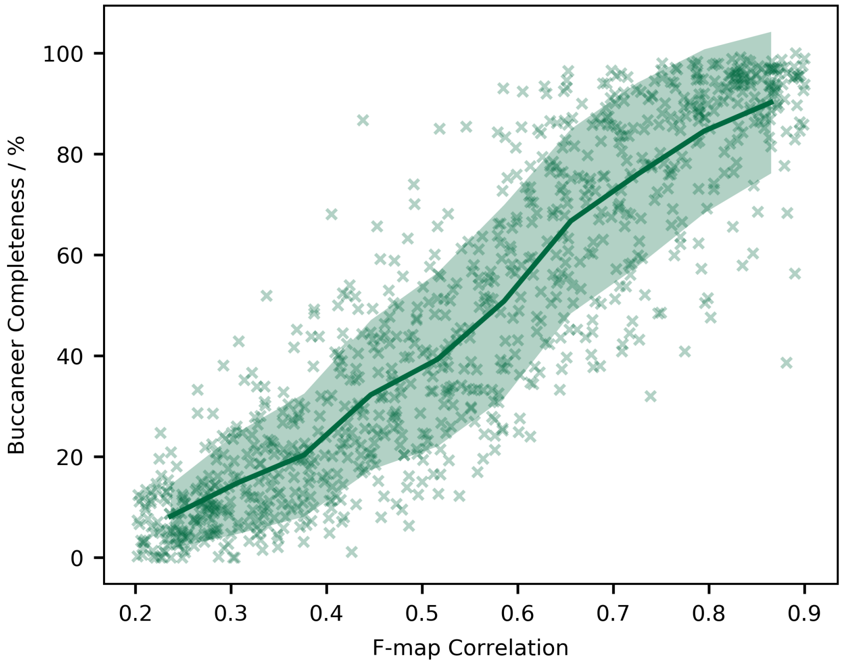

Figure 6: F-map correlation and completeness of the BUCCANEER model for 911 structures in the full reduced test set with resolutions 2.5 Å or better. Mean and standard deviation for 10 F-map correlation bins are overlaid.

can be adjusted to suit the needs of the user, such as the number of structures, resolution range, validation thresholds, number of models and the similarity of models.

An example test set was created that contains 15532 MR models across 1800 structures with resolutions between 1 Å and 3.5 Å. Hopefully this set is large enough that the program only needs to be repeated for more specialist needs. The full test set could be useful for assessing the performance of molecular replacement programs. However, most of the models do not produce correct solutions that are needed for testing model building and phase refinement programs.

Two reduced test sets were derived by selecting one model per structure. The easy reduced test set has 639 structures with resolutions between 1 and 2.5 Å where the model has an F-map correlation between 0.7 and 0.95. These should all be cases where automated model building works well so they will be useful for comparing model completion algorithms, where the aim is to replace routine manual model building tasks that need to be done after automated building has finished. The full reduced test set contains 1351 structures between 1 and 3.5 Å resolution with F-map correlations between 0.2 and 0.9, which is useful for assessing the performance of automated model building in more challenging cases.

The BUCCANEER pipeline from CCP4i was tested on both reduced test sets. As expected, performance was better at high resolution and with good quality phases. The pipeline runs for 5 cycles by default and, although this is sufficient for most of the easy cases, performance on some of the more difficult cases will be improved with more cycles.

This issue has been addressed by recent improvements to the CCP4i2 [22] pipeline, which will be discussed in a future publication.

5 Availability

All the data relating to this publication are available at:

https://doi.org/10.15124/44145f0a-5d82-4604-9494-7cf71190bd82

This includes the test set creation program and other scripts, the unreduced test set with multiple models per structure, and both reduced test sets. The test set creation program is also available on GitHub at https://github.com/paulsbond/create-mr-set.

6 Acknowledgements

The authors would like to thank Ronan Keegan for his advice on searching for structural homologues using GESAMT and Huw Jenkins for help with PHASER.

This work was supported by the White Rose BBSRC DTP in Mechanistic Biology (BB/M011151/1) and BBSRC grant BB/S005099/1.

References

1. Foadi, J. *et al.* A flexible and efficient procedure for the solution and phase refinement of protein structures. *Acta Crystallographica Section D* **56,** 1137–1147 (2000).
2. Perrakis, A., Morris, R. & Lamzin, V. S. Automated protein model building combined with iterative structure refinement. *Nature structural & molecular biology* **6,** 458–463 (1999).
3. Langer, G., Cohen, S. X., Lamzin, V. S. & Perrakis, A. Automated macromolecular model building for X-ray crystallography using ARP/wARP version 7. *Nature protocols* **3,** 1171–1179 (2008).
4. Berman, H. M. *et al.* The Protein Data Bank. *Nucleic Acids Research* **28,** 235–242 (2000).
5. Winn, M. D. *et al.* Overview of the *CCP*4 suite and current developments. *Acta Crystallographica Section D* **67,** 235–242 (2011).
6. Yang, H. *et al.* *DCC*: a Swiss army knife for structure factor analysis and validation. *Journal of Applied Crystallography* **49,** 1081–1084 (2016).

7. Chen, V. B. *et al. MolProbity*: all-atom structure validation for macromolecular crystallography. *Acta Crystallographica Section D* **66,** 12–21 (2010).

8. Kleywegt, G. J. *et al.* The Uppsala Electron-Density Server. *Acta Crystallographica Section D* **60,** 2240–2249 (2004).

9. DW. Using BLASTClust to Make Non-redundant Sequence Sets. *NCBI News.* `ncbi.nlm.nih.gov/Web/Newsltr/Spring04/blastlab.html` (2004).

10. French, S. & Wilson, K. On the treatment of negative intensity observations. *Acta Crystallographica Section A* **34,** 517–525 (1978).

11. Murshudov, G. N. *et al. REFMAC*5 for the refinement of macromolecular crystal structures. *Acta Crystallographica Section D* **67,** 355–367 (2011).

12. Kovalevskiy, O., Nicholls, R. A., Long, F., Carlon, A. & Murshudov, G. N. Overview of refinement procedures within *REFMAC*5: utilizing data from different sources. *Acta Crystallographica Section D* **74,** 215–227 (2018).

13. Krissinel, E. Enhanced fold recognition using efficient short fragment clustering. *Journal of Molecular Biochemistry* **1,** 76–85 (2012).

14. Krissinel, E. & Uski, V. Desktop and Web-based Gesamt Software for Fast and Accurate Structural Queries in the PDB. *Journal of Computer Science Applications and Information Technology* **2,** 1–7 (2017).

15. Higgins, D. G. & Sharp, P. M. CLUSTAL: a package for performing multiple sequence alignment on a microcomputer. *Gene* **73,** 237–244 (1988).

16. Bunkóczi, G. & Read, R. J. Improvement of molecular-replacement models with *Sculptor*. *Acta Crystallographica Section D* **67,** 303–312 (2011).

17. McCoy, A. J. *et al. Phaser* crystallographic software. *Journal of Applied Crystallography* **40,** 658–674 (2007).

18. Cowtan, K. The *Buccaneer* software for automated model building. 1. Tracing protein chains. *Acta Crystallographica Section D* **62,** 1002–1011 (2006).

19. Cowtan, K. Fitting molecular fragments into electron density. *Acta Crystallographica Section D* **64,** 83–89 (2008).

20. Read, R. J. & McCoy, A. J. A log-likelihood-gain intensity target for crystallographic phasing that accounts for experimental error. *Acta Crystallographica Section D* **72,** 375–387 (2016).

21. Alharbi, E., Bond, P. S., Calinescu, R. & Cowtan, K. Comparison of automated crystallographic model-building pipelines. *Acta Crystallographica Section D* **75,** 1119–1128 (2019).

22. Potterton, L. *et al. CCP4i2*: the new graphical user interface to the *CCP*4 program suite. *Acta Crystallographica Section D* **74,** 68–84 (2018).
